# Are All Child-to-Parent Violence Profiles Associated with Exposure to Family Violence? Findings from a Sample of Spanish Adolescents

**DOI:** 10.3390/healthcare11121710

**Published:** 2023-06-11

**Authors:** Esther Calvete

**Affiliations:** Department of Psychology, Faculty of Health Sciences, University of Deusto, 48007 Bilbao, Spain; esther.calvete@deusto.es; Tel.: +34-9413-9000

**Keywords:** child-to-parent violence, exposure to family violence, profiles, knowledge structures, social information processing

## Abstract

Exposure to family violence (EFV) is proposed as a relevant antecedent of child-to-parent violence (CPV). However, both previous research and practitioner experience suggest that not all cases of CPV involve EFV. This study aimed to identify profiles of adolescents according to their degree of involvement in CPV and their EFV. A sample of 1647 adolescents (mean age = 14.30, *SD* = 1.21; 50.5% boys) completed measures of CPV, witnessing family violence, victimization by parents, permissive parenting, parental warmth, and several measures of cognitive and emotional characteristics. Latent profile analyses based on measures of CPV and family characteristics supported a four-profile solution. Profile 1 (82.2%) consisted of adolescents with very low scores on both CPV and exposure to family violence. Profile 2 (6.2%) was characterized by medium scores on psychological CPV and high EFV. Profile 3 (9.7%) was characterized by severe psychological CPV and very low EFV. Profile 4 (1.9%) included adolescents with the highest scores on CPV, including physical violence, and high EFV. These profiles were found to differ from each other according to several cognitive and emotional variables of the adolescents. Therefore, not all CPV profiles were associated with a history of EFV. The obtained profiles have implications for interventions.

## 1. Introduction

In recent decades, there has been a growing interest in children’s aggressions towards their parents. Child-to-parent violence (CPV) has been defined as repeated behaviors of physical, psychological (verbal or nonverbal), or economic violence directed at parents or those who take their place [1]. Although the data on the frequency of CPV are mixed, research suggests that it is a relatively common problem in Western societies. For example, an important review found prevalence rates between 33 to 93% for psychological CPV and between 5 and 21% for physical CPV in community samples [2]. In the case of Spain, according to the Report of the State Attorney General’s Office, 4699 cases of CPV were opened in 2020 [3].

Regarding sex, the vast majority of studies conducted with community samples indicate that boys and girls exhibit similar levels of violence against their fathers and mothers [4,5]. However, there may be differences in the type of violence. Specifically, psychological violence tends to be more common in girls, while physical violence tends to be more common in boys [5]. This may partially explain why studies with samples of offenders find more cases of boys than girls [6,7,8]. Regarding the age of adolescents, a study on the longitudinal trajectory of CPV in adolescents concluded that the frequency of aggressions increased until the age of 15 and then began to decrease [9]. This is consistent with data obtained from judicial samples indicating a peak between the ages of 14 and 17 [2].

According to a recent review [10], several theoretical models have been used to explain the development of CPV, including behavioral, cognitive, psychodynamic, and psychosocial models. For example, based on the behavioral model, Bandura’s social learning theory helped to explain how, in some cases of CPV, the adolescent imitates aggressive behaviors previously observed in the family environment [11]. From the cognitive model, the role of social information processing [12,13] has been highlighted as an antecedent to the perpetration of CPV [14]. From the systemic perspective, the nonviolent resistance model emphasizes the cycles of action and reaction that are established between the adolescent and his or her parents [15]. In addition to these models, there are other perspectives that integrate many of the mechanisms proposed by the aforementioned theories. For example, Simmons et al. adapted the social-ecological perspective to CPV [2]. This perspective is based on a systemic approach to reality and includes numerous factors that can contribute to the development of CPV, which are organized into several subsystems: ontogenetic, microsystem, exosystem, and macrosystem.

### 1.1. Family Variables Associated with CPV

Numerous studies have focused on identifying family variables that may increase the risk of CPV. These variables include exposure to family violence, permissive parenting, and a lack of parental warmth (for reviews, see [2,16]). Of all these variables, exposure to family violence has probably received the most attention [17,18,19,20]. Indeed, one of the most consistent findings during the several decades of the study of CPV is that it often coexists with other forms of family violence, including child maltreatment and domestic violence perpetrated against mothers and other family members.

Although many studies have examined the role of generic measures of exposure to family violence, most studies have specifically differentiated between direct and indirect family victimization [16,21]. Direct victimization refers to a child directly experiencing forms of neglect, physical and emotional maltreatment, and sexual abuse. Indirect victimization occurs when the child observes violence perpetrated against the mother and other family members. However, it is necessary to clarify that this differentiation is somewhat forced, because there is a consensus among experts that exposure to violence toward the partner should be included as a form of the direct emotional maltreatment of children [16]. When the mother in a family is abused, children can be seriously affected by witnessing the violence. Therefore, witnessing violence against the mother is also regarded as a form of direct victimization. In a recent meta-analysis [21], both forms of victimization, child maltreatment and witnessing violence, were found to be associated with an increased likelihood of developing CPV [21]. Moreover, these results were quite stable as a function of various moderators, such as the type of CPV (physical or psychological) and sample (offenders or community).

However, not all studies have consistently identified the presence of exposure to family violence in the development of CPV. For example, in a recent study, psychological and physical child abuse were associated with adolescents’ violence toward their parents, but child exposure to intimate partner violence was not associated with CPV [22]. Moreover, qualitative research on adolescent perpetrators of CPV also indicates that exposure to family violence, among other factors, is present at the origin of many but not all cases of CPV. In a study in which adolescents receiving treatment in a specialized CPV center were interviewed in depth, 73% of the adolescents reported having been victims of family abuse, generally of a physical nature, and 57% claimed to have witnessed violence against their mothers at home [23], but the rest had not been exposed to family violence. Finally, data obtained from psychotherapeutic work with families who had experienced CPV revealed varied scenarios, many of which did not include exposure to other forms of family violence but involved other family characteristics, such as permissiveness and a lack of limits and parental warmth [24].

The role of permissiveness and a lack of limits in discipline has been examined in several studies. Cottrell and Monk (2004) found that permissiveness led to a power role reversal, whereby children perceived that the positive reinforcements they gained through their inappropriate behaviors outweighed the punishments [25]. Thus, children learned that their violent behavior served to achieve their goals. This is consistent with what has been found in clinical work with families, where parents often encounter difficulty in establishing rules and limits and request that professionals help them to achieve this [24,26]. However, the role of discipline strategies employed by parents in the development of CPV is currently uncertain, as the results obtained from numerous studies have been inconsistent. Thus, in a review, the authors concluded that the relationship between discipline and CPV is complex [2].

Another important family characteristic associated with CPV is a lack of parental warmth [27,28,29,30,31]. In a longitudinal study over three years, a lack of parental warmth was the parenting aspect that best predicted the increase in aggression against both the mother and father [27]. These types of findings have led to the suggestion that affectivity and the quality of family relationships are the most important aspects to prevent CPV [31].

### 1.2. Adolescents’ Characteristics Associated with CPV

In addition to family characteristics, numerous studies have focused on identifying the emotional and cognitive characteristics of adolescents who engage in CPV. These characteristics are greatly influenced by family experiences (e.g., exposure to violence and permissiveness [32]). According to relevant theoretical models of aggression in humans, such as the general aggression model [32] and the social information processing model [12,13], important individual variables (e.g., knowledge structures and cognitive and emotional processing) play an important role as antecedents of aggressive behavior. Knowledge structures consist of organized elements of past behaviors and experiences that form a relatively cohesive and persistent body of knowledge that guides one’s subsequent perception and appraisal of the world [32]. Previous research has indicated the importance of three knowledge structures for explaining aggressive behavior: the justification of violence, narcissism, and mistrust/hostility structures [33,34]. The justification of violence refers to normative beliefs about the social appropriateness of aggression [35], including the idea that the use of aggression is justified and leads to positive outcomes for the individual. Narcissism involves the belief that one is superior to others, deserving of special rights and privileges. Mistrust/hostility consists of the expectation that others will hurt, abuse, humiliate, or take advantage of an individual, and it usually involves the belief that the harm is intentional [33]. A few studies have examined the role of these knowledge structures in CPV (for a review, see [36]). For example, several quantitative studies with community samples have found relationships between grandiosity, mistrust/hostility [27,37], the justification of violence [27,38,39], and CPV.

The abovementioned knowledge structures are hypothesized to guide information processing in social settings. In the case of CPV, a few studies have examined the cognitive and emotional components that precede the perpetration of aggressive behavior from a proximal perspective. Calvete et al. (2014) [14] adapted the information processing model [12,35], and the emotional components subsequently added to the model [40,41,42], to CPV. In their study, they found that when adolescents who perpetrate CPV experience ambiguous social situations with their parents, they attribute hostile intentions to their parents, experience anger, select hostile or revenge goals, expect positive outcomes from aggressive behavior, and do not feel empathy towards their parents [14]. Subsequently, other studies have provided additional evidence for the role of these variables in CPV [18,38,39].

Finally, numerous studies have found that adolescents who perpetrate CPV often exhibit emotional and behavioral problems. Many of these problems derive in part from the family characteristics and cognitive and emotional styles described above. For instance, externalizing problems such as substance abuse and overall aggressive behavior is frequent among adolescents who perpetrate CPV [4,8,25,43,44]. It has also been found that these adolescents often experience symptoms of distress and depression [43,45,46].

### 1.3. The Current Study

Previous research on CPV has paid considerable attention to the role that certain family characteristics may play in the origin and development of this problem. In this context, exposure to family violence, both in the form of child maltreatment and witnessing violence against the mother, has motivated numerous studies. In general, the results of these studies agree, showing that exposure to family violence is a risk factor for CPV [21], but the results are not always consistent for the various modalities of exposure (i.e., child maltreatment and witnessing [22]). Moreover, both quantitative and qualitative research and clinical case reports indicate that many adolescent perpetrators of CPV have neither experienced child maltreatment nor observed violence in their homes [23,24]. Thus, the present study addresses the potential existence of different CPV profiles, which may be characterized by different family circumstances. For example, it has been proposed that CPV could stem from permissive parenting styles that fail to set limits on children’s behavior [24] or from a lack of positive warmth in parenting [27,30,31].

Most previous studies have adopted a perspective based on variable-centered analyses and examined the association between modalities of CPV (e.g., psychological and physical) and family characteristics (e.g., exposure to family violence and permissiveness). In contrast, this study employed a different approach based on person-centered analysis: latent profile analysis (LCA). The conceptual model on which the use of this analysis was based was the existence of profiles of individuals characterized by different combinations of traits, which may be interconnected with each other. Thus, LCA aims to split data into subclasses of two or more homogeneous groups [47,48,49,50]. The current study aimed to explore the profiles of adolescents according to their involvement in CPV and family characteristics (child maltreatment, the witnessing of family violence, a lack of limits, and parental warmth). This approach could be useful in determining the profiles of adolescent perpetrators of CPV in different family contexts (e.g., in families where other forms of family violence coexist and in families where there are different circumstances such as permissiveness) and thus inform the design of practice-based interventions targeted toward the specific profiles that emerged from the analysis [50].

In addition, in order to better understand the resulting adolescent profiles, this study examined whether the profiles differed according to important adolescent variables found in previous research to be relevant to CPV: sex, age, knowledge structures related to violence, and social information processing that precedes aggression against parents. The study adopted an exploratory perspective, so no prior hypotheses were established about the characteristics of the profiles that would emerge. However, interest was focused on determining whether there was a profile of adolescents involved in CPV who had not been exposed to family violence and on exploring whether this profile differed from the others in terms of the study variables.

## 2. Materials and Methods

### 2.1. Participants

A sample of 1647 adolescents aged 11 to 18 years (mean age = 14.30, SD = 1.21; 50.5% boys) participated in the study. They were contacted through 22 high schools (9 public and 13 private) in Bizkaia, Spain. Regarding family situation, 78.9% of the adolescents lived with both parents, 16.5% with the mother, 13.8% with the father, 1.4% in other contexts (homes and foster families), and 26 adolescents did not provide this information. As for the country of origin, 89.4% were Spanish, and the rest came from other countries (7.9% South American countries, 1% African countries, 1.4% other European countries, and 0.3% Asian countries). Finally, according to parental occupation data [51], the socio-economic levels of the participants were as follows: 16.6% low, 10.9% medium-low, 21.8% medium, 33.3% medium-high, and 17.4% high.

### 2.2. Measures

CPV was assessed using the Child–Parent Aggression Questionnaire (CPAQ, [5]). The CPAQ consists of 20 parallel items: 10 referring to aggressions against the father and 10 against the mother. There are four items for moderate psychological aggressions (e.g., taking money without permission, doing something to annoy); three for severe psychological aggressions (e.g., insulting, threatening to hit without doing so); and three for physical aggressions (e.g., hitting the father or mother with something that could hurt or kicking him or her). The participants were invited to indicate how often they had engaged in each of the behaviors in the past year using the following four-point scale: 0 (never); 1 (has occurred one or two times); 2 (has occurred three to five times); and 3 (has occurred six or more times). For this study, following the procedure used in other studies [5], total scores were obtained for moderate psychological aggression against the mother, severe psychological aggression against the mother, physical aggression against the mother, and the equivalent scores against the father. The CPAQ has displayed excellent psychometric properties in several samples of Spanish adolescents [5,52] and in other Spanish-speaking countries, such as Mexico [53]. In this study, for aggressions against mothers, the ordinal α coefficients were 0.73, 0.70, and 0.90 for moderate psychological aggression, severe psychological aggression, and physical aggression, respectively, and for aggression against fathers, they were 0.72, 0.70, and 0.92.

Exposure to family violence was assessed using an adapted version of the Exposure to Violence Scale [54,55]. This scale includes both victimization and witnessing violent acts in the family. The subscale of direct victimization in the family consists of three items (e.g., “How many times have you been hit or physically harmed at home?”). The witnessing family violence subscale includes five items referring to witnessing intimate partner violence (e.g., “How many times have you observed at home how a man physically assaults his partner?”). Each item was answered on a five-point scale (0 = never to 4 = every day). In this study, the ordinal α was 0.87 for direct victimization in the family and 0.90 for witnessing family violence.

Parental warmth was assessed using the affection and communication subscale of the Parenting Style Scale [56]. This scale includes eight items for each parent (e.g., “My father/mother enjoys talking things over with me”), which are rated on a four-point scale (1 = not at all true to 4 = completely true). The ordinal α was 0.96.

Permissiveness was assessed using the permissive style subscale of the Young Parenting Inventory [57], which describes a parenting style whereby parents accept that children do not take responsibility for their behaviors and are treated as holders of special rights. This subscale consists of four parallel items (four for each parent), describing behaviors such as “It was too permissive in many ways” and “It gives me little control and norms”. This scale has been previously used in other samples, including clinical and batterer samples [58,59]. Items were answered using a four-point scale (1 = not at all true to 4 = completely true). The ordinal α was 0.92.

Violence-related knowledge structures were assessed with the justification of violence subscale of the Irrational Beliefs Scale for Adolescents (IBSA; [60]) and the subscales of narcissism and mistrust/hostility of the Young Schema Questionnaire-3 ([61]; adapted to Spanish by [62]). The justification of violence subscale of the IBSA consists of nine items that reflect the idea that aggression is appropriate in a variety of situations (e.g., “Sometimes you have to hit others because they deserve it”) and that aggression enhances self-esteem and helps to maintain status among peers (e.g., “Being good at fighting is something to be proud of”). Each item is rated on a four-point Likert-type scale ranging from 1 (not at all true) to 4 (completely true). The narcissism and mistrust/hostility subscales consist of five items each. The narcissism or grandiosity subscale refers to the belief that one is superior to other people and entitled to special rights and privileges (e.g., “I’m special and shouldn’t have to accept many of the restrictions placed on other people”). The mistrust/hostility subscale (5 items) describes an individual’s expectation that others will hurt, abuse, humiliate, lie, or take advantage of them (e.g., “I feel that people will take advantage of me”) and usually involves the belief that the harm is either intentional or the result of negligence. Items are rated using a six-point Likert-type scale ranging from 1 (completely untrue of me) to 6 (describes me perfectly). The ordinal α coefficients were 0.62 and 0.71 for narcissism and mistrust/hostility, respectively.

Social information processing was assessed by means of the Social Information Processing in Child–Parent Conflicts Questionnaire [14]. The questionnaire includes three scenarios describing conflicts with parents (e.g., “It’s Saturday night and you really would like to go to a party with your friends but you have no money. You ask your parents for some but they refuse, saying that they already gave you money this week”). The adolescents had to imagine each scenario and respond to the items to assess five components: hostile attribution, which includes attributions of negative intentions and positive emotions in parents (two items per scenario); anger (one item per scenario); aggressive response selection, including both physical and psychological aggression (two items per scenario); the anticipation of positive consequences for aggressive behavior (one item per scenario); and empathy or the anticipation of negative consequences for parents (one item per scenario). Items were answered using a five-point response scale ranging from 0 (not at all) to 4 (to a great extent). The ordinal α coefficients were 0.82, 0.79 0.95, 0.94, and 0.95, respectively, for hostile attribution, anger, aggressive response selection, positive consequences, and empathy.

Behavioral problems were assessed using a brief version of the externalizing scale of the Youth Self-Report (YSR; [63]). This scale includes eight items from the Aggressive Behavior and the Rule-Breaking Behavior subscales of the YSR (e.g., being disobedient at school, starting fights, and using alcohol and/or drugs). Items are rated from 0 (not true) to 2 (very or often true). The ordinal α was 0.85.

Finally, depressive symptoms were assessed with the shortened version [64] of the Centre for Epidemiological Studies Depression Scale (CES-D; [65]). This version consists of 10 items, which are answered on a four-point Likert scale ranging from 0 (rarely or none of the time) to 3 (most or all of the time). Sample items are “I felt that I could not shake off the blues even with help from my family or friends” and “I felt sad”. The ordinal α was 0.76.

### 2.3. Procedure

Recruitment was carried out via schools. Initially, 32 schools in Bizkaia (Basque Country) were invited to participate. The headmasters of 22 of the schools agreed to participate. After their approval to participate in the study, parents of the adolescents in these schools were notified and given the option to refuse their child’s participation in the study. None of the parents refused their child’s participation. Then, the adolescents were invited to participate, and all of them agreed. The participants completed both the interventions and study measures in their classroom. No identifying data were included to protect the identity of the participants. The study was approved by the Ethics Committee of the University of Deusto.

### 2.4. Statistical Approach

Latent profile analysis with MPLUS 8.8 [66] was used to explore adolescent profiles based on involvement in aggressive behavior against parents, exposure to family violence, and parenting styles. Models were estimated using the maximum likelihood estimator. Initially, a single-profile LPA model was estimated to serve as a comparative baseline for models with more than one profile. Then, successive models were estimated, increasing the number of profiles by one. The resulting solutions were examined to determine whether they were statistically and conceptually superior to the previous model [49]. Several criteria were followed to determine the optimal number of profiles [47,48,49]: the Akaike Information Criteria (AIC), Bayesian Information Criteria (BIC), Sample-Size-Adjusted BIC (SSABIC), Lo–Mendell–Rubin Likelihood Ratio Test (LMRLRT), Bootstrap Likelihood Ratio Test (BLRT), and entropy. AIC, BIC, and SSABIC are approximate fit indices wherein lower values indicate superior fit [49]. Higher values of entropy suggest better fit and are considered good at 0.80 and above. The LMRALR and BLRT compare whether a k-profile solution fits better than a k-1-profile solution and considers this to be the case when they are statistically significant. Additionally, mean posterior probabilities were examined, which provide information on the effectiveness of a given model in classifying individuals into their most likely classes. A value of 0.70 or higher is considered adequate for mean posterior probabilities [48]. Finally, in addition to the previous indicators, the number of participants within each profile and the meaning and usefulness of the profile solution obtained were assessed.

To determine the differences between adolescents belonging to each profile in the variables of the study, an analysis of variance (ANOVA) was performed with pairwise comparisons (Bonferroni method, *p* < 0.05). Moreover, a multiple testing correction was performed using the Benjamini–Hochberg false discovery rate method [67]. Chi-square tests were performed to determine sex differences in the profiles. In addition, path analysis, which controlled for overlaps between variables, was performed to identify which variables were associated with CPV profiles. The comparative fit index (CFI), Tucker/Lewis fit index (TLI), root mean square error of approximation (RMSEA), and standardized root mean square residual (SRMR) were used to evaluate the fit of the model. CFI and TLI values of 0.95 or greater and RMSEA and SRMR values of 0.05 or lower indicated that the model adequately fit the data. Missingness was examined with the Little’s Missing Completely at Random (MCAR) test [68], which was statistically significant (χ^2^ (618) = 1132, *p* < 0.001). Thus, multiple imputation (*N* = 100 samples) was used for the analysis.

## 3. Results

Table 1 displays the main descriptive statistics of the variables and the correlation coefficients between them. As observed, there was a high correlation between the mother aggression scales and their parallels against the father (coefficients ranging between 0.60 and 0.77). There was also a high correlation between the two forms of exposure to family violence. Both exposure to violence and permissiveness were positively associated with the CPV subscales, while parental warmth was negatively associated. All knowledge structures and information-processing components were positively associated with CPV. The exception was the empathy subscale, which was not significantly associated with most of the CPV subscales. Finally, both externalizing behaviors and depressive symptoms were associated with the CPV subscales.

### 3.1. Profiles of CPV

Models ranging from one to five profiles were estimated based on the measures of CPV and family characteristics. Table 2 displays the main characteristics of the models. Consistently, all models of two or more profiles showed the existence of a profile that included most of the sample (range: 1160–1612) and a very small number of adolescents (range: 30–35). All solutions showed very high entropy values. The three- and four-profile solutions showed the best indicators: lower AIC, BIC, and SSABIC values than the solutions with fewer profiles and statistically significant LMRLRT and BLRT values. However, the solution of four profiles was considered optimal given the composition of the profiles obtained, as described below. After deciding that the optimal number was four profiles, posterior probabilities were used to assign each participant to a single profile. The mean posterior probabilities assigned to the profiles were 0.95, 0.99, 0.95, and 1.00, respectively, for profiles 1, 2, 3, and 4. Finally, the number of participants in each profile was found to be sufficient, except for the profile with 31 adolescents.

**Figure 1:**
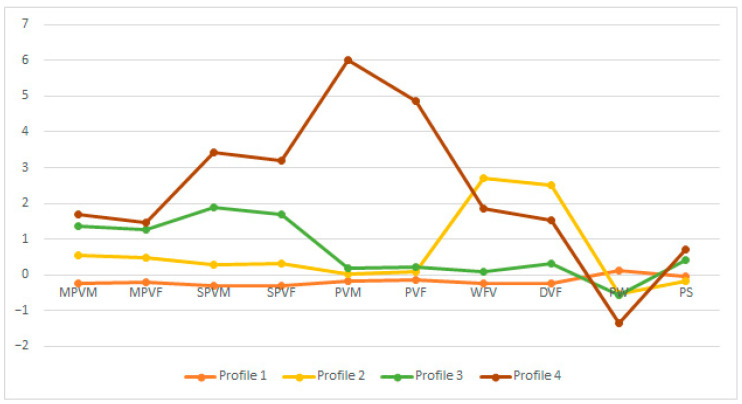
Profiles of adolescents according to their involvement in child-to-parent violence and family variables. Note: MPVM = moderate psychological violence against mother; MPVF = moderate psychological violence against father; SPVM = severe psychological violence against mother; SPVF = severe psychological violence against father; PVM = physical violence against mother; PVF = physical violence against father; WFV = witnessing family violence; DVF = direct victimization in the family; PW = parental warmth; PS = permissive style.

Table 3 displays the differences between the profiles for all the variables. The effect sizes for the differences were large for all the variables (ηp 2 > 0.14), except for parental warmth and a lack of limits in parenting. In order to facilitate the interpretation of the profiles, the standardized scores are shown in Figure 1. Profile 1 was the most numerous (82.2%, *n* = 1354) and consisted of adolescents with very low scores on both CPV and exposure to family violence. Profile 2 (6.2%, *n* = 102) was characterized by medium scores on moderate psychological CPV and very high scores on both forms of exposure to family violence. Profile 3 (9.7%, *n* = 160) was characterized by high scores on severe psychological CPV and very low exposure to family violence. Profile 4 (1.9%, *n* = 31) included adolescents with the highest scores on CPV, including physical aggression, and high exposure to family violence.

### 3.2. Differences between Profiles in terms of Other Variables

In general, the percentages according to sex were quite similar, except in the case of profile 3, which was significantly more frequent in girls than in boys (12.4% girls vs. 6.9% boys; χ^2^ (1) = 14.29, *p* < 0.011). There was no association between adolescent CPV profiles and socioeconomic level (χ^2^(12) = 8.44, *p* = 0.750). The four profiles were compared in terms of scores on knowledge structures, social information processing, externalizing problems, depressive symptoms, and age. Table 4 displays the ANOVA results. The effect size was high (>0.14) for aggressive response selection and moderate for hostile attribution, the anticipation of positive consequences for aggression, and externalizing problems. The effect size was small for the other variables. Table 4 presents the results of the post hoc comparisons.

Next, a path analysis was conducted using paths between the adolescent variables and membership of profiles 1, 2, and 4. Since dummy variables were used for the profiles, the results indicated an association with these profiles compared to profile 3, which acted as a reference. Multiple imputation (100 samples) was used, so confidence intervals for the coefficients are included. Since this model was saturated, a more parsimonious model was estimated in which some non-significant paths were eliminated. The fit indices of this model were excellent: χ^2^ (10, *N* = 1647) = 15, *p* = 0.141, RMSEA = 0.017 (90% CI [0.000 to 0.034]), CFI = 0.997, TLI = 0.989, SRMR = 0.005. Table 5 displays the coefficients of the path analysis. According to the results, in comparison with profile 4, profile 3 was associated with less aggressive response selection in conflicts with parents and less anticipation of positive consequences for these aggressive responses. However, this group was associated with more experiences of anger in conflicts and more depressive symptoms than profile 4. There were many differences with respect to profile 2. Compared to this group, which was characterized by exposure to family violence, profile 3 was associated with older age and being female. It was also associated with lower hostility, both as a knowledge structure and in the processing of information preceding aggressive behavior, and with less anger in conflicts with parents. However, profile 3 was associated with higher levels of the justification of violence and the selection of aggressive behavior in conflicts with parents. Finally, in comparison to profile 1, profile 3 was associated with higher scores for hostile attribution, the selection of aggressive behavior, depression, and externalizing problems.

## 4. Discussion

The first objective of this study was to explore the existence of different adolescent profiles according to their involvement in CPV and other family characteristics. Among these family characteristics, special prominence was given to exposure to family violence, given the interest in this variable in previous research [2,16,21]. The results supported a solution consisting of four profiles as the most appropriate. These profiles and their implications are described below.

Profile 1 encompassed most of the adolescents in the sample and was characterized by almost no involvement in CPV. This profile showed the lowest levels in all modalities of CPV and in exposure to the two forms of family violence assessed in this study (direct victimization and witnessing). In addition, this profile was associated with the highest levels of parental warmth, a variable associated with less CPV in previous research [27,30,31], and relatively low scores on permissiveness [25].

Profile 2 included a small percentage of adolescents and was characterized by medium scores on psychological forms of CPV and very low scores on physical aggression. The most notable feature of this group was that it was characterized by the highest levels of exposure to both forms of family violence. Regarding other characteristics of parenting, this profile was associated with intermediate levels of parental warmth and low levels of permissiveness.

Profile 3 was of great interest in this study because it was characterized by low levels of exposure to family violence, though somewhat higher than those of the first profile. In terms of involvement in aggressive behavior towards parents, this profile showed high levels of psychological aggression—including severe aggression—and low levels of physical aggression—similar to those of the second profile. Profile 3 was associated with intermediate levels of parental warmth—similar to those of profile 2—and relatively high levels of permissiveness.

The last profile, profile 4, accounted for a very small percentage of the sample and was characterized by extremely high levels of all CPV modalities. Although exposure to family violence was not as high as in profile 2, the levels were higher than those for the other adolescent profiles. This profile also presented the lowest level of parental warmth and high levels of parental permissiveness.

Taken together, these profiles offer a more complete picture of the diverse scenarios associated with CPV. In the context of the large majority of adolescents who did not assault their parents, three profiles emerged with different degrees and modalities of aggression towards parents. These profiles differed in the severity of the aggressions as well as in the coexistence of CPV with forms of exposure to violence in the family. In fact, profile 3, which was characterized by high levels of psychological aggression, included adolescents who reported almost no experiences of direct victimization or witnessing violence against other family members.

Once these profiles were identified, the second objective involved exploring whether they also differed in terms of adolescent characteristics. Given the interest in profile 3, which was characterized by high levels of psychological CPV and low levels of exposure to family violence, it was used as a reference profile for comparing the others. In comparison with profile 1, which was characterized by a relative scarcity of CPV, profile 3 was associated with more hostile attribution and aggressive response selection in conflicts with parents, such as those caused when parents refused to give money to the adolescent or tried to set limits on their behavior, and higher levels of depressive symptoms and externalizing problems. However, the most relevant comparisons were those between profile 3 and the other two profiles involved in the perpetration of CPV. Compared to profile 2 (moderate psychological CPV combined with high exposure to family violence), profile 3 was associated more with girls than with boys, as well as with an older age, lower levels of mistrust/hostility and anger, and higher scores on the justification of violence and the selection of aggressive responses. It was also associated with lower scores on depressive symptoms and externalizing problems. This suggested that it was a predominantly female profile, with a clear justification of violence and predisposition to follow aggressive courses of action when faced with conflicts with parents. Importantly, however, this use of aggression appeared to be “cold” [69], as it was associated with lower scores on hostility and anger compared with profile 2. This finding is consistent with the idea that reasons to act aggressively against parents are diverse [70]. Finally, compared to profile 4, profile 3 was only statistically significantly associated with the less frequent selection of aggressive responses and anticipation of positive consequences for these responses and higher levels of anger and depressive symptoms. It should be noted that profile 4 showed the highest scores on most violence-related variables.

This study had several limitations. The first was related to the exclusive use of self-reports. Although self-reporting by adolescents is fundamental, especially when assessing aspects such as knowledge structures and the emotional and cognitive processing of information, it would be desirable to complement the measures with parents’ reports in order to have a less biased view of the CPV [71]. A second limitation was the use of a community sample, which resulted in a high number of adolescents clustered in the CPV-free profile. Although it has often been suggested that CPV is present in the general population and that reported cases represent only the tip of the iceberg [72], the study of profiles in samples of offenders would make it possible to obtain more cases of adolescents with high levels of CPV, especially of the physical type [52]. In this study, the fourth profile included very few participants, and therefore comparisons with this group should be made with caution.

In addition, although the study included numerous variables relevant to CPV, both familial and individual, it did not include other variables that might also be relevant in describing the various profiles. For example, this study did not include measures of some psychopathic traits, such as the callous unemotional trait, which according to other studies may also be associated with CPV [73]. Although the study included a general measure of behavioral problems, a specific measure of drug addiction should be included in future studies, as this variable has been found to be relevant for CPV [44]. Further, to avoid unduly increasing the time required for the participants to respond, in some cases it was not possible to use the complete questionnaires, and so only the subscales under investigation in this study were implemented.

It is also important to include measures of proactive and reactive aggression in the future. It may be the case that reactive and proactive types of behaviors discriminate between profiles 2 and 3 of adolescent perpetrators of CPV. Thus, future studies should examine whether profile 2, with a history of exposure to family violence, is characterized by higher levels of reactive aggressive behavior. This would be consistent with some of the traits detected in this group (i.e., greater hostility and anger), in agreement with previous studies [14,43,70]. On the other hand, it could be hypothesized that profile 3, without a history of exposure to family violence and with higher scores on the justification of violence and selection of aggressive responses, is characterized by a more instrumental use of aggression [14,43]

Despite these limitations, the study also had numerous strengths, such as the use of a large sample of adolescents and the inclusion of measures of variables relevant to CPV. Moreover, this study contributes to the literature on CPV by showing, through methodologies novel to the field, the existence of distinct CPV profiles. As Williams and Kibowski (2016) pointed out, latent profile analysis can be highly beneficial, because once participants are assigned to profiles based on their behaviors and characteristics, their profile membership can be used to inform practice-based policies and interventions targeted to specific profiles [50]. Specifically, the findings of this study could inform the development of preventive actions. The presence of exposure to family violence in two of the profiles of adolescents who perpetrated CPV places the focus back on the relevance of this factor and on the existence of a mechanism for the intergenerational transmission of violence in the context of the family [27,74,75]. This is important for detection and intervention. Professionals working with adolescents who perpetrate CPV should assess the co-occurrence of other forms of family violence, such as child abuse or violence against the mother by her partner, in order to implement the necessary interventions. Similarly, professionals working with female victims of gender violence who have children should promote preventive psychoeducational actions to prevent the vicarious learning of violence.

Finally, the existence of a profile of adolescents characterized by high levels of aggressive CPV in the absence of forms of direct and indirect victimization in the family suggests the need to establish other preventive strategies. In particular, the results of the study, while still preliminary, suggest that parental education on less permissive parenting and discipline strategies could be beneficial. Further, intervention programs, such as Coping Power, which teach adolescents non-aggressive ways of handling conflict and challenge knowledge structures that justify the use of violence, could contribute to the prevention of CPV [76].

## 5. Conclusions

In conclusion, and as an answer to the question that motivated this study, the results indicated the existence of different profiles according to the adolescents’ involvement in CPV. Moreover, they showed identified a profile of adolescents who perpetrated high levels of psychological CPV and who had not been exposed to violence in the family context. This profile was mostly characterized by females raised in families with relatively high levels of permissiveness. Compared to the other profiles associated with psychological CPV, this profile was mainly characterized by less severe emotional experiences (i.e., fewer symptoms of depression, less anger and hostility) and by a predisposition to justify the use of violence and to select aggressive options in conflicts with parents.

## Figures and Tables

**Table 1:** Correlation coefficients between variables and descriptive statistics.

	1	2	3	4	5	6	7	8	9	10	11	12	13	14	15	16	17	18	19	20
1. Moderate psychological aggression against mothers	1																			
2. Moderate psychological aggression against fathers	0.77	1																		
3. Severe psychological aggression against mothers	0.60	0.46	1																	
4. Severe psychological aggression against fathers	0.46	0.52	0.76	1																
5. Physical aggression against mothers	0.33	0.26	0.51	0.45	1															
6. Physical aggression against fathers	0.20	0.30	0.36	0.48	0.60	1														
7. Witnessing family violence	0.25	0.21	0.29	0.26	0.26	0.24	1													
8. Direct victimization in family	0.29	0.28	0.30	0.27	0.23	0.23	0.66	1												
9. Parental warmth	−0.25	−0.19	−0.29	−0.26	−0.20	−0.17	−0.19	−0.21	1											
10. Permissiveness	0.19	0.17	0.17	0.15	0.09 *	0.12	0.05 ^ns^	0.02 ^ns^	0.07 *	1										
11. Mistrust/hostility	0.23	0.21	0.18	0.18	0.10	0.10	0.19	0.22	−0.15	0.17	1									
12. Narcissism	0.27	0.23	0.23	0.19	0.13	0.11	0.20	0.20	−0.16	0.21	0.47	1								
13. Justification of violence	0.24	0.21	0.23	0.21	0.15	0.10	0.16	0.17	−0.22	0.13	0.31	0.41	1							
14. Hostile attribution	0.38	0.32	0.33	0.29	0.18	0.15	0.19	0.20	−0.35	0.10	0.24	0.26	0.29	1						
15. Anger	0.38	0.31	0.25	0.18	0.07 *	0.04 ^ns^	0.15	0.17	−0.19	0.08 *	0.11	0.20	0.23	0.49	1					
16. Aggressive response selection	0.40	0.36	0.47	0.42	0.40	0.28	0.23	0.21	−0.29	0.13	0.17	0.20	0.26	0.46	0.31	1				
17. Positive consequences	0.13	0.11	0.24	0.25	0.33	0.23	0.19	0.08 *	−0.17	0.09 *	0.07 *	0.08 *	0.17	0.24	0.07	0.55	1			
18. Empathy	−0.05 ^ns^	−0.04 ^ns^	−0.11	−0.09 *	−0.11	−0.09 *	−0.08 *	−0.06 *	0.14	−0.03 ^ns^	−0.05 ^ns^	−0.01 ^ns^	−0.09 *	−0.08 *	0.05 ^ns^	−0.16	−0.19	1		
19. Externalizing problems	0.37	0.36	0.37	0.35	0.21	0.17	0.27	0.25	−0.26	0.15	0.19	0.28	0.47	0.33	0.25	0.34	0.20	−0.07 *		
20. Depressive symptoms	0.23	0.22	0.23	0.22	0.14	0.16	0.25	0.29	−0.23	0.09	0.44	0.29	0.20	0.25	0.13	0.21	0.10	−0.07 *	0.37	1
Mean	10.71	10.40	0.58	0.47	0.12	0.11	0.21	0.36	40.58	20.57	20.21	20.62	20.37	10.00	20.13	0.27	0.15	30.42	0.88	10.24
SD	10.40	10.32	10.01	0.92	0.58	0.54	0.44	0.68	10.07	0.86	0.87	0.87	0.92	0.77	10.07	0.51	0.52	10.10	0.71	0.73

Note: all coefficients were statistically significant at *p* < 0.001, except those labeled with ^ns^ = no significant and * *p* < 0.05.

**Table 2:** Results of the latent profile analyses.

Fit Statistics	1	2	3	4	5
AIC	71,221.620	67,373.285	64,945.849	63,733.093	30,158.654
BIC	71,329.754	67,540.893	65,172.931	64,019.649	30,504.684
SSABIC	71,266.217	67,442.411	65,039.504	63,851.276	30,301.366
Entropy		1.00	0.966	0.969	0.920
LMRLRT (*p*)		3823.406 (*p* = 0.085)	2419.737 (*p* = 0.034)	1215.248 (*p* = 0.044)	658.398 (*p* = 0.608)
BLRT (*p*)		3870.334 (*p* < 0.001)	2449.436 (*p* < 0.001)	1230 (*p* < 0.001)	666.479 (*p* < 0.001)
Sample size of each profile	P1 = 1647	P1 = 35	P1 = 1,404	P1 = 1,355	P1 = 95
		P2 = 1612	P2 = 210	P2 = 103	P2 = 1160
			P3 = 33	P3 = 158	P3 = 276
				P4 = 31	P4 = 89
					P5 = 30

Note: P = profile; AIC = Akaike Information Criteria; BIC = Bayesian Information Criteria; SSABIC = Sample-Size-Adjusted BIC; LMRLRT = Lo–Mendell–Rubin Likelihood Ratio Test; BLRT = Bootstrap Likelihood Ratio Test.

**Table 3:** Comparisons between profiles in terms of family variables.

	Profile 1 M(SD)	Profile 2 M(SD)	Profile 3 M(SD)	Profile 4 M(SD)	*F*	*df*	*p*	ηp^2^	Differences between Profiles at *p* < 0.05
Moderate psychological CMV	5.49 (4.26)	9.87 (6.39)	14.49 (6.32)	16.19 (6.32)	230	3.1637	<0.001	0.30	P4 = P3 > P2 > P1
Moderate psychological CFV	4.46 (4.07)	8.20 (6.28)	12.23 (6.43)	13.35 (7.02)	171	3.1567	<0.001	0.25	P4 = P3 > P2 > P1
Severe psychological CMV	0.79 (1.30)	2.59 (2.80)	7.41 (3.22)	12.05 (5.21)	948	3.1636	<0.001	0.64	P4 > P3 > P2 > P1
Severe psychological CFV	0.61 (1.13)	2.23 (2.75)	6.02 (3.79)	10.16 (5.67)	612	3.1565	<0.001	0.54	P4 > P3 > P2 > P1
Physical CMV	0.09 (0.46)	0.42 (1.08)	0.70 (1.39)	10.89 (4.99)	1341	3.1637	<0.001	0.71	P4 > P3 = P2 > P1
Physical CFV	0.09 (0.56)	0.43 (1.65)	0.65 (1.59)	8.13 (6.13)	467	3.1565	<0.001	0.47	P4 > P3 = P2 > P1
Witnessing family violence	0.47 (1.04)	7.02 (3.14)	1.19 (1.68)	5.19 (4.58)	702	3.1612	<0.001	0.57	P2 > P4 > P3 > P1
Direct victimization in family	0.56 (1.17)	6.22 (2.49)	1.71 (2.23)	4.20 (3.18)	519	3.1614	<0.001	0.49	P2 > P4 > P3 > P1
Parental warmth	4.72 (1.00)	4.00 (1.15)	3.95 (1.15)	3.12 (1.07)	55	3.1589	<0.001	0.09	P1 > P2 = P3 > P4
Permissiveness	2.53 (0.84)	2.41 (0.89)	2.91 (0.95)	3.17 (0.83)	15	3.1585	<0.001	0.03	P3 = P4 > P1 = P2

Note: P = profile; CMV = child-to-mother violence; CFV = child-to-father violence.

**Table 4:** Comparisons between profiles in adolescents’ variables.

	Profile 1 M(SD)	Profile 2 M(SD)	Profile 3 M(SD)	Profile 4 M(SD)	*F*	*df*	*p*	ηp^2^	Differences between Profiles at *p* < 0.05
Mistrust/hostility	2.13 (0.84)	2.67 (0.92)	2.48 (0.89)	2.78 (0.79)	25	3.1642	<0.001	.04	P4 = P2 = P3 > P1
Narcissism	2.53 (0.82)	2.97 (1.09)	2.98 (0.85)	3.33 (0.93)	18	3.1643	<0.001	.05	P4 = P3 = P2 > P1
Justification of violence	2.28 (0.89)	2.56 (0.92)	2.82 (0.90)	3.24 (0.94)	19	3.1643	<0.001	.05	P4 = P3; P4 > P2; P2 = P3; P2 > P1
Hostile attribution	0.89 (0.69)	1.40 (0.89)	1.54 (0.89)	1.83 (0.93)	64	3.1635	<0.001	.10	P4 = P3, P4 > P2, P3 = P2, P1 < P2 P3 P4
Anger	2.01 (1.06)	2.60 (0.89)	2.72 (0.99)	2.48 (1.17)	31	3.1635	<0.001	.05	P1 < P2, P3; P2 = P3 = P4
Aggressive response selection	0.18 (0.39)	0.37 (0.57)	0.64 (0.62)	1.59 (1.06)	144	3.1639	<0.001	.21	P4 > P3 > P2 >P1
Positive consequences	0.10 (0.44)	0.26 (0.68)	0.23 (0.60)	1.33 (1.15)	66	3.1634	<0.001	.11	P4 > P2 = P3 > P1
Empathy	3.46 (1.08)	3.22 (1.27)	3.37 (1.07)	2.46 (1.19)	10	3.1631	<0.001	.02	P1 = P2 = P3 > P4
Depressive symptoms	1.16 (0.69)	1.70 (0.81)	1.51 (0.72)	2.05 (0.81)	76	3.1495	<0.001	.07	P1 < P2, P3, P4; P4 >
Externalizing problems	0.77 (0.62)	1.28 (0.73)	1.45 (0.75)	1.74 (1.18)	8	3.1500	<0.001	.13	P1 < P2, P3, P4; P4 > P2; P2 = P3; P3 = P4
Age	14.28 (1.20)	14.03 (1.29)	14.72 (1.26)	14.17 (1.13)	38	3.1642	<0.001	.02	P3 > P1, P2; P4 = P1, P2, P3

Note: P = profile.

**Table 5:** Regressive coefficients of the path analysis model.

					Confidence Interval	
	Estimate	S.E.	Est./S.E.	*p*-Value	Lower 2.5%	Upper 2.5%
Profile 1						
Mistrust/hostility	−0.05	0.03	−1.94	0.053	−0.047	0.000
Narcissism	−0.05	0.03	−1.99	0.046	−0.046	0.000
Justification of violence	0.028	0.03	0.99	0.324	−0.011	0.034
Hostile attribution	−0.10	0.03	−3.44	0.001	−0.074	−0.020
Aggressive response selection	−0.18	0.03	−6.14	<0.001	−0.183	−0.094
Anger	−0.05	0.03	−1.86	0.062	−0.036	0.001
Empathy	0.03	0.02	1.17	0.244	−0.006	0.025
Positive consequences for aggression	−0.01	0.03	−0.08	0.937	−0.040	0.037
Depressive symptoms	−0.06	0.03	−2.28	0.022	−0.060	−0.005
Externalizing problems	−0.22	0.03	−7.86	<0.001	−0.149	−0.089
Female	−0.04	0.02	−1.69	0.091	−0.065	0.005
Age	−0.01	0.02	−0.35	0.726	−0.016	0.011
Profile 2						
Mistrust/hostility	0.09	0.03	30.07	0.002	0.009	0.042
Narcissism	0.03	0.03	0.99	0.324	−0.008	0.024
Justification of violence	−0.10	0.03	−0.23	0.001	−0.042	−0.010
Hostile attribution	0.06	0.03	1.97	0.049	0.000	0.037
Aggressive response selection	0.07	0.03	−2.19	0.029	−0.065	−0.004
Anger	0.08	0.03	2.69	0.007	0.005	0.030
Empathy	−0.04	0.03	−1.59	0.111	−0.019	0.002
Positive consequences for aggression	0.04	0.03	1.20	0.231	−0.010	0.043
Depressive symptoms	0.08	0.03	2.49	0.013	0.005	0.044
Externalizing problems	0.12	0.03	3.77	<0.001	0.020	0.062
Female	−0.06	0.03	−2.32	0.021	−0.053	−0.004
Age	−0.07	0.02	−2.95	0.003	−0.024	−0.005
Profile 4						
Mistrust/hostility	−0.01	0.03	−0.31	0.756	−0.010	0.007
Narcissism	0.04	0.03	1.44	0.149	−0.002	0.015
Justification of violence	0.01	0.03	0.26	0.792	−0.007	0.009
Hostile attribution	−0.02	0.03	−0.78	0.435	−0.014	0.006
Aggressive response selection	0.28	0.03	9.07	<0.001	0.058	0.090
Anger	0.06	0.03	−2.18	0.030	−0.014	−0.001
Empathy	0.04	0.02	−1.76	0.079	−0.011	0.001
Positive consequences for aggression	0.14	0.03	5.11	<0.001	0.022	0.051
Depressive symptoms	0.07	0.03	2.40	0.016	0.002	0.023
Externalizing problems	0.03	0.03	0.99	0.324	−0.006	0.017
Female	−0.02	0.02	−0.72	0.475	−0.018	0.008
Age	−0.02	0.02	−0.76	0.450	−0.007	0.003

## Data Availability

Data are available under request.
